# Asymptomatic Bacteriuria among Pregnant Women in Addis Ababa, Ethiopia: Prevalence, Causal Agents, and Their Antimicrobial Susceptibility

**DOI:** 10.1155/2021/8418043

**Published:** 2021-07-17

**Authors:** Ketema Bizuwork, Haile Alemayehu, Girmay Medhin, Wondwossen Amogne, Tadesse Eguale

**Affiliations:** ^1^Aklilu Lemma Institute of Pathobiology, Addis Ababa University, P.O. Box 1176, Addis Ababa, Ethiopia; ^2^College of Health Sciences, Addis Ababa University, P.O. Box 1176, Addis Ababa, Ethiopia

## Abstract

Asymptomatic bacteriuria (ASBU) is an important health problem among pregnant women, particularly in low-income countries. This study aimed to estimate the prevalence of ASBU and potential risk factors among pregnant women attending antenatal care centers in Addis Ababa. It also aimed to identify causal bacterial pathogens and to assess their antimicrobial susceptibility. A health facility-based cross-sectional study was conducted from March to June 2019. Urine samples from a total of 281 pregnant women with no symptoms of urinary tract infection were tested for ASBU. Women whose urine samples carried greater than or equal to 10^5^ colony-forming units (CFU) of bacteria per milliliter of urine when grown on plate count agar were considered positive for ASBU. Bacterial pathogens were isolated from urine samples of women with ASBU using standard microbiological techniques. Antimicrobial susceptibility of isolates was investigated using the Kirby–Bauer disk diffusion method on Muller–Hinton agar plates. Of 281 pregnant women examined, 44 (15.7%) were positive for ASBU. Logistic regression analysis of the putative risk factors tested in the current study showed that none of them were significantly associated with the occurrence of ASBU (*p* > 0.05). The most frequently isolated bacterial species were *Escherichia coli* 17 (30.2%), *Proteus* 13 (23.2%), and *Enterococcus* 11 (19.6%). All of the *E. coli, Citrobacter*, and *Klebsiella* isolates and 84.6% of *Proteus* were resistant to ampicillin. All bacterial isolates were resistant to at least one of the antimicrobials tested. Resistance to three or more antimicrobials was detected in 15 (88.2%) of *E. coli*, 13 (100%) of *Proteus*, and 8 (72.7%) of *Enterococcus* isolates. Resistance to as many as 7 antimicrobials among *E. coli*, 8 antimicrobials among *Proteus*, and 7 antimicrobials among *Enterococcus* isolates was recorded. Detection of ASBU in a substantial number of pregnant women in this study warrants the need for a detailed study on possible risks of developing symptomatic urinary tract infection (UTI) and associated complications. Multidrug resistance to several antimicrobials was observed in the majority of bacterial isolates. Regular assessment of antimicrobial susceptibility of uropathogens to commonly prescribed antimicrobials and implementation of prudent use of antimicrobials are recommended.

## 1. Introduction

Asymptomatic bacteriuria (ASBU) is defined as the existence of bacteria in urine at a load of 10^5^ CFU/ml or more in the absence of clinical symptoms of urinary tract infection [1, 2]. Although both genders and all age groups are prone to ASBU, it is more common in women due to the proximity of female urethra to the anus which facilitates colonization of the periurethral area with bacteria from gastrointestinal tract [3]. Its occurrence in women increases directly with sexual activity and in women of child-bearing age [4]. Diabetic patients, women with low level of educational status, and those with history of urinary tract infection are reported to be at higher risk of developing ASBU [5]. Pregnant women are particularly prone to urinary tract infection and ASBU due to physiologic changes associated with pregnancy like smooth muscle relaxation, dilation of the ureters, and renal pelvis which favor bacterial multiplication [6]. In addition, a general decline in immunity during pregnancy predisposes women to infection [7].

Pregnant women with ASBU suffer from various adverse outcomes. For instance, 50% of pregnant women with ASBU were reported to develop symptomatic urinary tract infection manifested by pyelonephritis followed by high rate of intrauterine growth restriction leading to low birth weight infants, increased risk of preterm labor, pregnancy-induced hypertension, preeclampsia, amnionitis, and anemia [8, 9]. Systematic review and meta-analysis showed strong association of ASBU with both type 1 and type 2 diabetes [10]. A prospective observational study among women with diabetes and ASBU also reported increased risk of hospitalization for urosepsis [11].

Prevalence of ASBU among pregnant women varies across different countries, and it is more common in developing countries. In Africa, prevalence of 24.7% was reported from Nigeria [12], whereas in Ghana, prevalence of 5.5% was reported [13]. Prevalence of 21.2% and 18.8% was reported from north and south Ethiopia [14, 15]. The common bacterial species isolated from pregnant women with ASBU include *Escherichia coli*, *Klebsiella species*, *Enterococcus faecalis*, *Proteus*, and *Staphylococcus aureus* [16–18]. Potential complications associated with ASBU can be significantly decreased in pregnant women if treated with appropriate antimicrobials [19]. However, reports elsewhere show that pathogenic bacteria including uropathogens are developing resistance to antimicrobials, and multidrug resistant strains are emerging rapidly posing major threat [20]. Understanding the rate of occurrence of ASBU among pregnant women, factors associated with its occurrence, identification of bacterial pathogens involved, and their antimicrobial susceptibility is vital in advising clinicians on appropriate management of urinary tract infection and associated complications. Therefore, this study aimed to assess the occurrence of ASBU, risk factors associated with ASBU, bacterial pathogens involved, and their antimicrobial susceptibility among pregnant women attending antenatal care services in Addis Ababa, Ethiopia.

## 2. Materials and Methods

### 2.1. Study Area, Study Population, and Study Design

This study was conducted in Addis Ababa, the capital city of Ethiopia, from March to June 2019. The target population was pregnant women residing in Addis Ababa whereas source population involved all pregnant women attending antenatal care centers of the selected health facilities. The study participants were pregnant women who attended the antenatal care centers of selected hospitals during the study period and consented to be involved in the study. Health facility-based cross-sectional study design was employed to recruit pregnant women by simple random sampling method from antenatal care centers of two government hospitals, namely, Tikur Anbessa Specialized Hospital (TASH) and Zewditu Memorial Hospital (ZMH), and two private healthcare facilities: Anania Mothers and Children Specialized Medical Center and Hemen Maternal and Children's Specialty Medical Center located in Addis Ababa.

### 2.2. Sample Size Determination and Recruitment of Study Participants

The sample size was calculated using single population proportion formula based on the previous report of 21.2% of ASBU in the north Ethiopia [15]. With 95% confidence interval, 10% nonresponse rate, and 5% marginal error, the calculated sample size was 283. First, total sample size calculated was allocated into the study hospitals proportional to the number of pregnant women attending selected antenatal care centers. This was based on the total number of women who visited the respective health facilities during one month prior to the initiation of the study. Once clear explanation about the purpose of the study was given to each pregnant woman coming to antenatal care center for routine antenatal care services, they were requested to be involved in the study. Pregnant women excluded from the study were those <18 years of age, those with symptomatic urinary tract infection, those who took two or more glasses of fluid one hour before clinic attendance, those who were treated with antimicrobials during the last one week, and those with current symptoms of sexually transmitted infections.

### 2.3. Data and Sample Collection

Information about age, residence, marital status, educational status, monthly income, frequency of vaginal douching per day, history of urinary tract infection in the current pregnancy, past history for chronic diseases (diabetes mellitus, hypertension), history of sexually transmitted diseases, stage of pregnancy, and parity was collected by trained nurse from each woman individually through interview for analysis of potential predictors of ASBU. Current status of the pregnant women for HIV/AIDS and Hepatitis B surface antigen was reviewed from the recent information sheets of pregnant women's chart. The study participants were instructed to properly wash perineal area before sample collection. Then, a clean-catch midstream urine samples were collected into sterile containers and transported to Microbiology Laboratory of Aklilu Lemma Institute of Pathobiology in ice box within 3-4 hours of collection.

### 2.4. Urine Sample Processing and Pathogen Isolation

A loopful (10 microliters) of well-mixed urine sample was inoculated onto plate count agar (PCA) and grown for 24 h at 37°C. Number of colonies grown on each plate was counted to determine colony-forming units per milliliter (CFU/ml) of the urine sample. A pregnant woman with urine sample containing ≥10^5^ CFU/ml was considered as having ASBU [8]. For determination of the type of organisms involved, colonies from PCA plates were picked and inoculated to various selective media such as Eosin Methylene Blue (EMB) agar to differentiate *Escherichia coli*, *Klebsiella species*, and *Citrobacter* species; cysteine-lactose-electrolyte-deficient (CLED) medium to differentiate *Proteus* species; bile esculin agar and enterococcus agar to identify *Enterococcus* species after incubating for 24 hr at 37°C. In addition, urine samples from women with ASBU were also directly plated to selective and differential media (Mannitol salt agar, EMB, and CLED agar). Isolates were categorized into Gram-negative and Gram-positive based on potassium hydroxide (KOH) string test. Biochemical tests such as triple sugar iron agar, lysine iron agar, citrate, urease, indole, and catalase tests were employed to further identify bacterial species involved [21–23]. One confirmed colony of each bacterial isolate was grown overnight in tryptic soy broth and stored at −80°C in 20% glycerol until further testing.

### 2.5. Investigation of Antimicrobial Susceptibility of Bacterial Isolates

Antimicrobial susceptibility test was performed according to the Clinical Laboratory Standards Institute (CLSI) guideline using Kirby-Bauer disk diffusion method on Muller-Hinton agar plates (Oxoid, CM0337 Basingstoke, England) [24]. The following antimicrobials (Sensi-Discs, Becton, Dickinson and Company, Loveton, USA) and disc potencies were used: amoxicillin + clavulanic acid (Amc) (20/10 *μ*g), ampicillin (Amp) (10 *μ*g), cephalothin (Cf) (30 *μ*g), ceftriaxone (Cro) (30 *μ*g), ciprofloxacin (Cip) (5 *μ*g), gentamicin (Gm) (10 *μ*g), streptomycin (S) (10 *μ*g), sulfisoxazole (G), sulfamethoxazole + trimethoprim (Sxt) (23.75/1.25 *μ*g), tetracycline (Te) (30 *μ*g), chloramphenicol (C) (30 *μ*g), vancomycin (Va) (30 *μ*g), kanamycin (K) (30 *μ*g), erythromycin (Ery) (15 *μ*g), oxacillin (Ox) (1 *μ*g), clindamycin (Da) (2 *μ*g), and penicillin (P) (10 *μ*g). *Escherichia coli* ATCC 25922 and *Staphylococcus aureus* ATCC25923 were used as quality control organisms for Gram-negative and Gram-positive organisms, respectively. The interpretation of the categories of susceptible and resistant was based on the CLSI guidelines [24]. Isolates were regarded as multidrug resistant (MDR) when they were resistant to at least two or more antimicrobials belonging to different classes [25].

### 2.6. Ethical Consideration

Study was approved by the Institutional Review Board of Aklilu Lemma Institute of Pathobiology, Addis Ababa University (Ref. No. ALIPB IRB/022/2011/2019). Permission was obtained from administrative office of each of the study hospitals and healthcare specialty centers. The purpose of the study was clearly explained to each participant, and informed verbal consent was obtained before initiation of data and sample collection.

### 2.7. Data Analysis

Descriptive statistics: mean, standard deviation, frequency, and percentage were used to summarize different variables. Logistic regression was used to assess association of predefined independent variables with the binary outcome. *p* value less than 0.05 was considered as statistically significant.

## 3. Results

### 3.1. Prevalence of Asymptomatic Bacteriuria

Although we approached a total of 283 pregnant women, we were able to get complete information and urine samples only from 281 (99.3%) of them. Out of 281 cultured urine samples, 44 had greater than or equal to 10^5^ CFU/ml of bacteria, resulting in 15.7% prevalence of ASBU with no significant difference between pregnant women attending private healthcare institutions (*n* = 14; 18.0%) and government hospitals (*n* = 30; 14.8%). Forty-one (16.6%) married and 3 (8.8%) unmarried pregnant women were positive for ASBU with no statistically significant difference between the groups. Similarly, differences in monthly income, educational status, and age were not associated with the occurrence of ASBU (Table 1).

### 3.2. Association of Selected Factors with the Occurrence of ASBU

Frequency of vaginal douching OR = 1.83 (95% CI: 0.82, 4.07) and history of previous urinary tract infection in the current pregnancy OR = 1.23 (95% CI: 0.59, 2.58) were not significantly associated with the occurrence of ASBU. Similarly, history of chronic diseases, parity, stage of pregnancy, serology test status for human immunodeficiency virus and hepatitis virus, VDRL test status, and history of treatment for UTI in this pregnancy did not show statistically significant association with the occurrence of ASBU (Table 2).

### 3.3. Common Bacterial Pathogens Isolated from Pregnant Women with ASBU

A total of 56 bacterial pathogens were isolated from 44 pregnant women with ASBU of which 45 (80.4%) were Gram-negative. These include *E. coli* 17 (30.4%) followed by *Proteus* spp. 13 (23.2%), *Klebsiella* spp. 8 (14.3%), and *Citrobacter* spp. 7 (12.5%). The remaining 11 (19.6%) of the isolates were Gram-positive organisms belonging to *Enterococcus* spp. Among the 44 participants that were positive for ASBU, 15 (34.1%) were positive for more than one bacterial pathogen. The most frequent bacterial species that appeared jointly in a single pregnant woman's urine sample were *E. coli* and *Proteus.*

### 3.4. Antimicrobial Susceptibility of Bacterial Isolates

All of the *E. coli*, *Citrobacter*, and *Klebsiella* isolates and 84.6% of *Proteus* were resistant to ampicillin. Similarly, 70.6% of *E. coli*, 100% of *Citrobacter* and *Klebsiella* isolates, and 84.6% of *Proteus* isolates were resistant to cephalothin. High rate of resistance to other antimicrobials such as amoxicillin + clavulanic acid and sulfisoxazole was also recorded. Resistance to ceftriaxone was detected in 17.6%, 28.6%, 37.5%, and 38.4% of *E. coli*, *Citrobacter*, *Klebsiella*, and *Proteus* isolates, respectively. All of the *Enterococcus* isolates were resistant to penicillin, whereas 90.9% and 72.7% of the isolates were resistant to oxacillin and amikacin, respectively. Relatively low level of resistance to ciprofloxacin was observed in all species of bacteria isolated in the current study (Table 3).

Resistance to 3 or more antimicrobials was detected in 15 (88.2%), 13 (100%), and 8 (72.7%) of *E. coli*, *Proteus*, and *Enterococcus* isolates, respectively. The common resistance pattern detected among *E. coli* isolates was resistance to G-Cf-Am-Amc in 6 (35.6%) of the 17 isolates. Three* E. coli* isolates in the current study were resistant to 7 antimicrobials tested. One (7.7%) of the *Proteus* isolates was resistant to 8 antimicrobials and one (12.5%) of the *Klebsiella* isolates was resistant to seven antimicrobials. Out of the eleven *Enterococcus* isolates tested, eight (72.7%) were resistant to three or more antimicrobials tested. Three of the *Enterococcus* isolates obtained from the pregnant women were resistant to 7 antimicrobials (Table 4).

## 4. Discussion

Presence of high load of bacteria in the urine of pregnant women is reported to lead to complications like pyelonephritis and septicemia and may also result in low birth weight and still birth [9]. The current study showed that the prevalence of ASBU among pregnant women attending antenatal care services in Addis Ababa was 15.7% which is in line with 18.8% prevalence reported from south Ethiopia [14], but lower than the prevalence reported from Northern Ethiopia (21.2%) [26] and Nigeria (24.7%) [12]. It is however higher than the prevalence reported from Ghana (5.5%) [13] and North West Ethiopia (9.8%) [27]. The possible reason for such difference could be due to variation in care during urine collection, difference in socioeconomic status, and genital hygienic practices among pregnant women from different backgrounds [28, 29].

Although previous reports showed association of occurrence of ASBU with increased maternal age, multiparity [28, 30], sexual activity [13], and past history of urinary tract infection among pregnant women [31], our finding did not show association of ASBU with any of these factors. The most frequently isolated bacterial uropathogens in this study was *E. coli* 17 (30.6%), which is in agreement with previous studies elsewhere [15, 31, 32]. However, it is contrary to the study from India that reported *S. aureus* as the most common pathogen detected among pregnant women with ASBU [33]. None of the pregnant women with ASBU in the current study were positive for *S. aureus* unlike the previous studies [15, 32]. Previous studies also showed that *S. aureus* bacteriuria is unusual except in patients with predisposing conditions for ascending colonization such as history of urinary obstruction, urinary catheter, recent urological surgical procedures, malignancy, and recent hospitalization [34–36].

*Proteus* spp., 13 (23.2%), was the second most frequently isolated bacteria among pregnant women in this study. Similar occurrence of *Proteus* spp. was reported previously in 20% of pregnant women with ASBU in a study conducted in Ghana and 9.1% in north Ethiopia [13, 15]. The enzyme urease produced by *Proteus* spp. hydrolyzes urea present in excess amount in the urine producing ammonia that leads to increased pH of the urine which favors precipitation of stones and other UTI complications [37].

Only Gram-positive bacterium isolated in the current study was *Enterococcus* spp., 11 (19.6%), which is in line with previous reports from Ghana (26.7%) and Kenya (1.8%) [13, 32], whereas previous studies from Ethiopia on pregnant women with ASBU did not report *Enterococcus* spp. [14, 15]. This difference may be due to differences in socioeconomic characteristics, season of the study, and clinical and background characteristics. In addition, not targeting *Enterococcus* spp. when culturing bacteria from urine samples in the previous studies might have also contributed to the observed differences.

Overall, rate of occurrence of resistance to antimicrobials tested in the current study is variable when compared to previous studies. Some of the isolates were resistant to ciprofloxacin and ceftriaxone, antimicrobials commonly used for treatment of various infectious pathogens. Compared to isolates reported from pregnant women with ASBU previously in Ethiopia, high rate of resistance to ampicillin was recorded in the current study among Gram-negative isolates. For instance, only 68.8% of isolates of *E. coli* were resistant to ampicillin [14] in previous study from south Ethiopia, whereas 100% resistance was recorded in the current study. On the other hand, the same previous study reported 43.8% resistance to ceftriaxone while only 17.6% resistance was detected in the current study. A study from north Ethiopia reported 100% resistance to ampicillin and 5.2% resistance to ceftriaxone, the third-generation cephalosporin [15]. A study from Kenya reported high rate of resistance (74.1%) to another third-generation cephalosporin (cefotaxime) among *E. coli* isolates compared to 117.6% resistance to ceftriaxone in the current study [32]. Resistance to sulfamethoxazole + trimethoprim was detected only in 5.9% of *E. coli* isolates in the current study whereas resistance to as high as 47.4% and 82.2% was reported in previous studies in Ethiopia [14, 15]. However, none of the *E. coli* isolates from study in Uganda were resistant to sulfamethoxazole + trimethoprim [38]. High level of resistance to penicillin, oxacillin, and clindamycin was also detected in *Enterococcus* isolates in this study. Recent study from Uganda reported 50% resistance to ciprofloxacin and 100% resistance to amoxicillin + clavulanic acid, whereas in the current study, only 18.2% and 54.5% resistance was detected, respectively [38]. Such variation may be attributed to differences in frequency of antimicrobial use, difference in regulation of antimicrobial use in study areas, irrational use of antimicrobials such as sell without prescription, and self-medication [39].

Most of the bacterial isolates in the current study were resistant to two or more antimicrobials tested. Previous study from Ambo town, central Ethiopia, also reported resistance to 2 or more antimicrobials in all of the bacterial isolates from pregnant women with symptomatic urinary tract infection and those with ASBU [40]. Detection of high level resistance to ampicillin in *E. coli* and *Citrobacter* spp. in the current study shows the need for use of alternative antimicrobials to treat infections caused by these organisms in the area. The finding of this study revealed that 27.3% of the *Enterococcus* spp. isolates were resistant to vancomycin, which is lower than the finding from India that reported over 80% resistance [41]. This may be due to differences in sociodemographic and background characteristics, differences in awareness level of transmission and/or prevention of infectious bacteria, and/or differences in extent, frequency, and/or manner of antimicrobial use.

Some of the isolates detected in the current study were multidrug resistant to about 6-8 antimicrobials in the case of Gram-negative organisms, and some of the *Enterococcus* isolates were resistant to 7 antimicrobials suggesting the need for rapid action on prudent use of antimicrobials in the study area. Even though the data generated from this study gives significant scientific evidence on status of ASBU in the study area, the fact that the number of pregnant women involved in the study was small and the interview was based on self-report rendered recall bias and social desirability bias. Hence, it may not be generalized to the wider community.

## 5. Conclusions

ASBU in pregnant women was recorded in this study which may lead to possible risk of developing symptomatic UTI and associated complications. High rate of MDR to antimicrobials commonly used to treat UTI infection has been recorded in bacterial isolates. Regular assessment of antimicrobial susceptibility of uropathogens to commonly prescribed antimicrobials instead of empirical therapy and implementation of prudent use of antimicrobials are recommended.

## Figures and Tables

**Table 1:** Association of demographic characteristics with ASBU in pregnant women attending antenatal care services in Addis Ababa, Ethiopia (*N* = 281).

Variables	No. tested	+ve for ASBU	COR (95% CI)	AOR (95% CI)	*p* value
Type of health facility	No. (%)				
Private	78	14 (18)	1.03 (0.50, 2.12)	0.62 (0.23, 1.66)	0.34
Government	203	30 (14.8)	^*∗∗*^		

Age					
18–24	74	9 (12.2)	1.41 (0.46, 4.00)	2.31 (0.63, 8.50)	0.23
25–34	166	28 (16.9)	0.78 (0.30, 201)		
35–49	41	7 (17.1)	^*∗∗*^		

Marital status					
Married	247	41 (16.6)	0.49 (0.14, 1.67)	0.52 (0.13, 2.05)	0.35
Unmarried	34	3 (8.8)	^*∗∗*^		

Educational status					
Illiterate	39	9 (23.1)	0.49 (0.19, 1.25)	0.57 (0.14, 2.25)	0.64
Grades 1–12	133	20 (15)	0.79 (0.38, 1.63)		
≥College	109	15 (13.8)	^*∗∗*^		

Income per month (ETB)					
≤2000	101	19 (18.8)	0.70 (0.36, 1.34)	0.87 (0.39, 1.92)	0.73
>2000	180	25 (13.9)	^*∗∗*^	^*∗∗*^	

ETB: Ethiopian birr, COR: crude odds ratio, AOR: adjusted odds ratio, CI: confidence interval.^*∗∗*^Reference.

**Table 2:** Association of background and clinical characteristics with ASBU in pregnant women attending antenatal care services in Addis Ababa, Ethiopia (*N* = 281).

Variables	No. tested	Positive for ASBU (%)	COR (95% CI)	AOR (95% CI)	*p* value
Frequency of vaginal douching per day					
≤Two times	117	14 (12)	1.65 (0.83, 3.27)	1.83 (0.82, 4.07)	0.14
≥Three times	164	30 (18.3)	^*∗∗*^		

History of chronic disease					
Yes	52	10 (19.2)	0.86 (0.39, 1.96)	0.84 (0.34, 2.07)	0.70
No	229	34 (14.8)	^*∗∗*^		

Parity					
Primipara	183	27 (14.8)	1.21 (0.63, 2.36)	1.14 (0.45, 2.88)	0.78
Multipara	98	17 (17.3)	^*∗∗*^		

Stage of pregnancy					
First trimester	103	19 (18.4)	0.77 (0.35, 1.66)	0.90 (0.35, 2.34)	0.83
Second trimester	90	12 (13.3)	1.13 (0.48, 2.63)		
Third trimester	88	13 (14.8)	^*∗∗*^		

Serology test status					
+ve for HIV/AIDS	13	3 (23.1)	0.61 (0.16, 2.30)	0.33 (0.75, 1.48)	0.15
+ve for HiBsAg	14	2 (14.3)	1.09 (0.23, 5.05)		
−ve for both	254	39 (15.4)	^*∗∗*^		

VDRL test status					
Positive	18	2 (11.1)	3.32 (0.43, 25.63)	3.00 (0.39, 27.77)	0.27
Negative	236	42 (17.8)	^*∗∗*^		

History of treatment for UTI in this pregnancy					
Yes	89	13 (14.6)	1.23 (0.59, 2.58)	1.51 (0.62, 3.67)	0.36
No	192	31 (16.1)	^*∗∗*^	^*∗∗*^	

Note: history of chronic disease includes diabetes mellitus (DM), hypertension (HPT), and renal calculi (RC). HIV/AIDS: human immune-deficiency virus/acquired immunodeficiency syndrome, HiBsAg: hepatitis B surface antigen, VDRL test: venereal disease (syphilis antibody) test, no.: number. ^*∗∗*^Reference.

**Table 3:** Antimicrobial susceptibility of bacteria isolated from pregnant women with ASBU attending antenatal care services in Addis Ababa, Ethiopia (*N* = 56).

Bacterial isolates	No. of antimicrobials tested and (%) resistant											
	Te	Sxt	S	Gm	Am	Cro	Cip	Cf	C	An	Amc	G
*E. coli* (*N* = 17)	3 (17.6)	1 (5.9)	4 (23.5)	2 (11.8)	17 (100)	3 (17.6)	1 (5.9)	12 (70.6)	1 (5.9)	1 (5.9)	13 (76.4)	9 (53)
*Citrobacter* spp. (*N* = 7)	1 (14.3)	1 (14.3)	4 (57.1)	0 (0.0)	7 (100)	2 (28.6)	0 (0.0)	7 (100)	0 (0.0)	3 (42.9)	6 (85.7)	6 (85.7)
*Klebsiella* spp. (*N* = 8)	0 (0.0)	2 (25)	2 (25)	1 (12.5)	8 (100)	3 (37.5)	1 (12.5)	8 (100)	1 (12.5)	1 (12.5)	8 (100)	3 (37.5)
*Proteus* spp. (*N* = 13)	3 (23.1)	1 (7.7)	3 (23.1)	1 (7.7)	11 (84.6)	5 (38.4)	0 (0.0)	11 (84.6)	2 (15.4)	2 (15.4)	12 (92.3)	4 (30.8)
*Enterococcus* spp. (*N* *=* 11)	Va	P	Ery	Cip	C	Cro	K	Cf	Ox	Da	Amc	Gm
	3 (27.3)	11 (100)	3 (27.3)	2 (18.2)	3 (27.3)	2 (18.2)	1 (9.1)	6 (54.5)	10 (90.9)	8 (72.7)	6 (54.5)	0 (0.0)

Te: tetracycline, Sxt: sulfamethoxazole + trimethoprim, S: streptomycin, Gm: gentamicin, Cro = ceftriaxone, Cip: ciprofloxacin, Cf: cephalothin, C: chloramphenicol, An: amikacin, Amc: amoxicillin + clavulanic acid, Am: ampicillin, Va: vancomycin, P: penicillin, Ery: erythromycin, K: kanamycin, Ox: oxacillin, Da: clindamycin, and G: sulfisoxazole.

**Table 4:** Antimicrobial resistance pattern of bacterial pathogens isolated from pregnant women (*N* = 56).

Bacterial species	Health facility	Resistance pattern	No. with R-pattern	No. of antimicrobials to which isolate is R
*E. coli* (17)				
	TASH (06)	Am,Te, S, Gm, Cro, C, An	1	7
		G, Cf, Amc, Am	5	4
	ZMH (09)	Cip, Te, Sxt, Gm, Cro, Am	1	6
		S, Am	2	2
		G. Cf, Am, Amc	6	4
	Private (02)	Cro, G, Te, S, Cf, Amc, Am	2	7

*Proteus* spp. (13)				
	TASH (04)	Te, Sxt, S, Gm, G, Am, Cf, An	1	8
		Cf, Amc, Am	3	3
	ZMH (08)	Cro, C, Am, Amc, Cf	5	5
		Te, S, G	3	3
	Private (01)	Cro, Cf, An, Am, Amc	1	5

*Enterococcus* spp. (11)				
	TASH (02)	P, Ox, Da, Cip, Cro, Cf, Amc	2	7
	ZMH (05)	P, Da, C, Va, Amc, Cf,	3	6
		Ery, Da, Ox, P	2	4
	Private (04)	P, Ox, Da, Cf, Amc, Ery, K	1	7
		P, Ox	3	2

*Klebsiella* spp. (8)				
	TASH (04)	S, C, Cf, Amc,Gm, Cip, An	1	7
		Cf, Amc, Cro	3	3
	ZMH (03)	Sxt, G, Cro, Cf, Amc	3	5
	Private (01)	Cf, Amc, S	1	3

*Citrobacter* spp. (7)				
	TASH (02)	S, G, Cf, An, Am, Amc	2	6
	ZMH (04)	S, Cro, Cf, Am, Amc, Te	1	6
		G, Am, Cf	3	3
	Private (1)	S, G, Cro, Cf, Am, An, Sxt, Amc	1	8

R: resistant, R-pattern: resistance pattern, Te: tetracycline, Sxt: sulfamethoxazole + trimethoprim, S: streptomycin, Gm: gentamicin, Cro: ceftriaxone, Cip: ciprofloxacin, Cf: cephalothin, C: chloramphenicol, An: amikacin, Amc: amoxicillin + clavulanic acid, Am: ampicillin, Va: vancomycin, P: penicillin, Ery: erythromycin, Ox: oxacillin, DA: clindamycin, G: sulfisoxazole, An: amikacin, TASH: Tikur Anbessa Specialized Hospital, and ZMH: Zewditu Memorial Hospital.

## Data Availability

All the data generated from this work are included within the manuscript.

## Abbreviations

ASBU: Asymptomatic bacteriuria
CFU: Colony-forming unit
CLSI: Clinical Laboratory Standards Institute
MDR: Multidrug resistant
PCA: Plate count agar
UTI: Urinary tract infection.
